# Peptide-Based Delivery Systems: Selected Insights into Cell-Penetrating Peptides

**DOI:** 10.1021/acschemneuro.6c00266

**Published:** 2026-06-03

**Authors:** Hussan Adam, Sara Abdollahi, Othman Al Musaimi

**Affiliations:** † School of Pharmacy, 5994 Newcastle University, Newcastle upon Tyne NE1 7RU, U.K.; ‡ Translational and Clinical Research Institute, Faculty of Medical Sciences, Newcastle University, Newcastle upon Tyne NE2 4HH, U.K.; § Department of Chemical Engineering, Imperial College London, London SW7 2AZ, U.K.; ∥ Orthogonal Peptides Limited, London SW7 2AZ, U.K.

**Keywords:** cell-penetrating peptides (CPPs), blood–brain barrier delivery, peptide-mediated transport, intracellular drug delivery, CNS therapeutics, peptide-based nanomedicine

## Abstract

The blood–brain barrier (BBB) is a highly selective biological interface that regulates molecular transport between the bloodstream and the central nervous system (CNS). Its primary function is to protect the brain from harmful substances while maintaining neural homeostasis. This regulatory capacity is attributed to its complex, multilayered structure, comprising nonfenestrated endothelial cells, astrocytic end-feet, pericytes, specialized transporters, and efflux pumps, along with dynamic mechanisms that adapt to physiological changes. Despite its essential protective role, the BBB presents a significant obstacle to the delivery of therapeutics targeting CNS disorders, limiting the ability of many drugs to reach their intended sites of action. Numerous invasive and noninvasive strategies have been explored to enhance CNS drug delivery; however, many are hindered by limitations such as low efficacy, poor specificity, and immunogenicity. Peptides, as endogenous short chains of amino acids, offer a promising alternative due to their high biocompatibility, ease of synthesis, and structural versatility. Their cationic charge facilitates interactions with negatively charged proteoglycans on cell surfaces, while their amphiphilic nature enhances membrane permeability, enabling efficient cellular uptake and translocation. These properties make peptide-based vectors particularly suitable for transporting diverse therapeutic cargoes across biological barriers. This review discusses the structural characteristics, origins, and classifications of peptide vectors, as well as the mechanisms underlying their cellular internalization. It further evaluates their advantages and current limitations in clinical applications, and highlights emerging strategies aimed at optimizing peptide-mediated delivery across the BBB.

## Graphical abstract

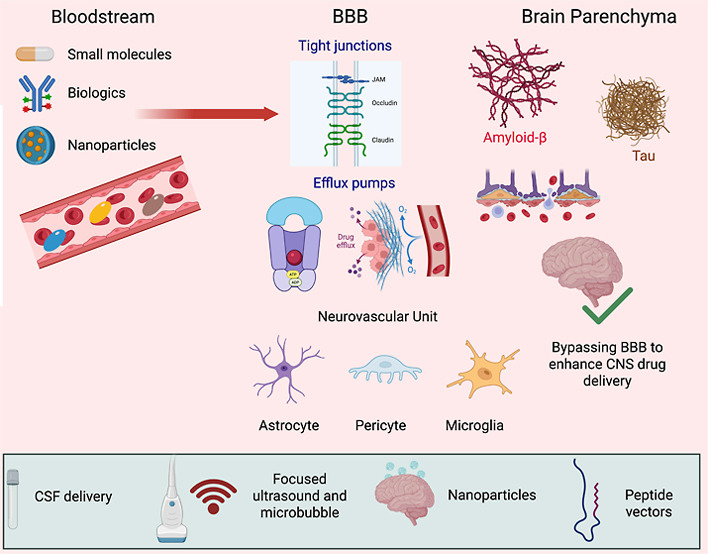

## 1 Introduction

The BBB is a dynamic physical, immunological, and metabolic interface that serves as a highly specialized microvascular system acting as a critical semipermeable boundary between the CNS and systemic circulation.^1–4^ It tightly regulates the bidirectional transport of molecules between the bloodstream and the brain parenchyma via endothelial cells (ECs),^5,6^ a process essential for maintaining cerebral homeostasis.^7^ For instance, the controlled influx and efflux of ions such as Na^+^, K^+^, Ca^2+^, and Cl^–^ through specific transporters is crucial for proper neuronal function.^8^ The transport of essential nutrients occurs through selective carriers expressed on the luminal surface of ECs, thereby protecting the brain from both endogenous and exogenous harmful substances. Notably, some transporters actively efflux xenobiotics and therapeutic agents back into the circulation.^9^ Among these, *P*-glycoprotein (*P*-gp) is a key efflux pump that constitutes an important component of the BBB’s protective mechanism, yet presents a significant challenge for CNS drug delivery.^9^ Consequently, approximately 90% of small molecules and nearly all large molecules are unable to cross the BBB.^10^

The concept of the BBB dates back to 1885, when Paul Ehrlich observed that systemically administered dyes failed to stain the CNS in animal models, suggesting restricted permeability.^11^ Subsequent investigations by Lewandowsky (1900), Goldman (1913), and Stern and Gautier (1918, 1921), along with advances in electron microscopy, confirmed both the existence and structural characteristics of the BBB.^12^

Structurally, the BBB is composed of a monolayer of continuous ECs characterized by the absence of fenestrations, high electrical resistance, and the presence of tight junctions (TJs) and adhesion proteins that ensure strong intercellular connectivity (Figure 1).^13^ These ECs are closely associated with astrocytes; a type of glial cell whose end-feet provide structural support and enhance barrier integrity while regulating nutrient supply to neurons.^14^ Astrocytes also play a metabolic role by storing glucose as glycogen and converting it to lactate when required, a process known as the astrocyte–neuron lactate shuttle.^14^ Owing to their complex morphology, astrocytes form intricate networks with neurons, contributing to higher brain functions such as learning and memory.^15,16^ Recent studies using genetically modified mouse models have further identified specific subtypes of perivascular astrocytes involved in regulating endothelial barrier integrity.^17^

**Figure 1:**
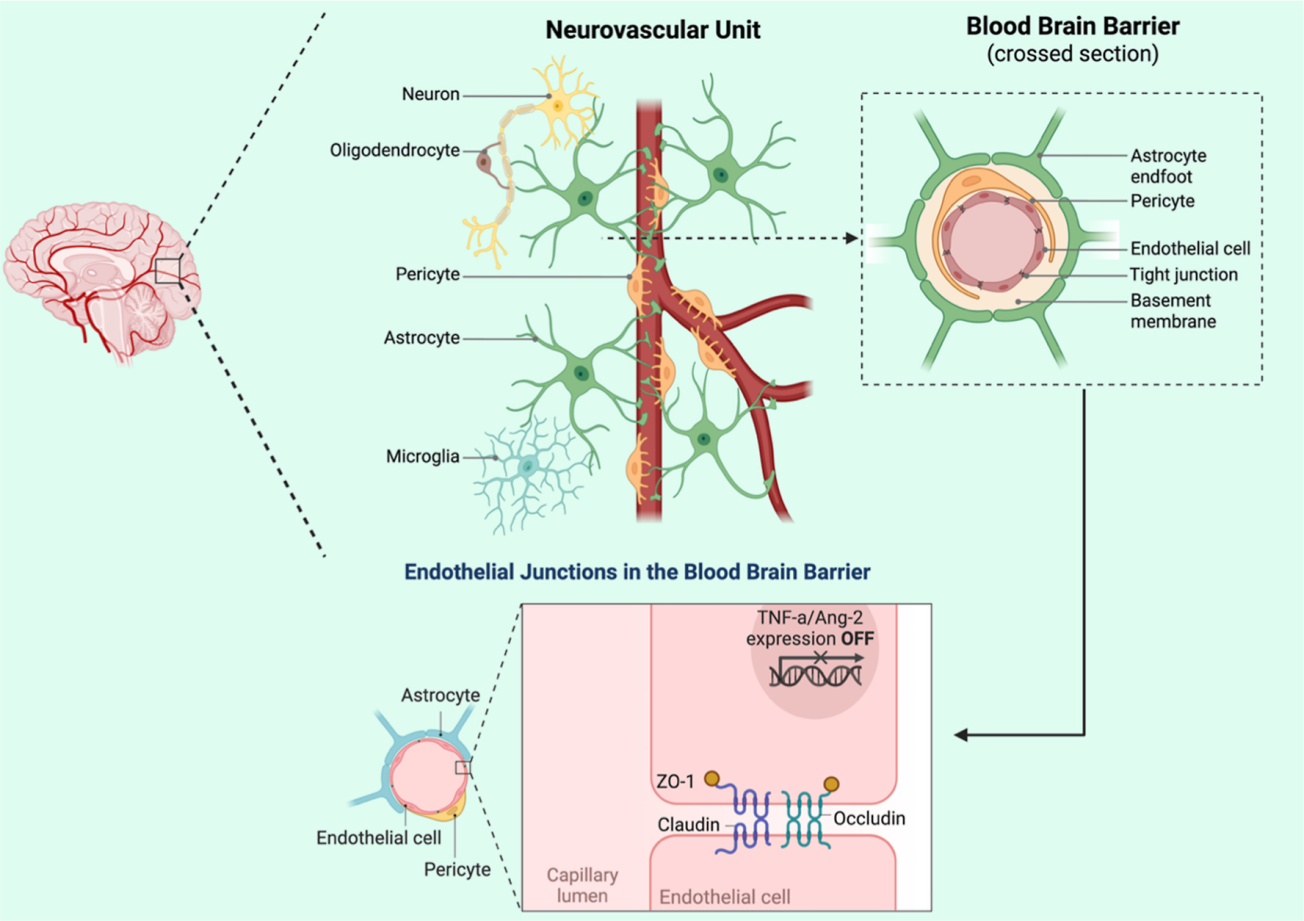
BBB composition, neurovascular unit, and endothelial junctions. Created using www.biorender.com.

Pericytes represent another critical component of the BBB. These mural cells envelop approximately 90% of the abluminal surface of microvessels and maintain close contact with ECs.^18^ They play essential roles in angiogenesis and in preserving BBB integrity.^19^ Collectively, ECs, astrocytic end-feet, pericytes, neurons, and associated cells form the neurovascular unit (NVU), which coordinates vascular and neuronal functions.^20,21^ In addition, the BBB possesses a specialized immune environment that includes resident microglia and a limited number of peripheral immune cells that can traverse the barrier under tightly regulated conditions.^22,23^ This controlled immune surveillance is necessary to minimize inflammation and prevent neuronal damage.^22,23^

This review aims to examine the major challenges associated with the delivery of therapeutics to the CNS, evaluate current strategies developed to overcome these barriers along with their advantages and limitations, and highlight examples of peptide-based vectors as a promising approach for enhancing drug delivery across the BBB.

## 2 Challenges in CNS Drug Delivery

Despite substantial progress in understanding the CNS, the development of effective CNS therapeutics remains highly challenging. High attrition rates of CNS drug candidates, particularly during Phase II and Phase III clinical trials, are frequently attributed to inadequate penetration of the BBB.^24^ The selective nature and restricted permeability of the BBB continue to represent major limitations in CNS drug development.^24^ Transport across the BBB occurs through several distinct mechanisms. Oxygen, other gases, and small lipophilic molecules (typically <400 Da) can cross the BBB via passive diffusion. In contrast, essential nutrients such as amino acids, carbohydrates, and certain hormones are transported through specific carrier-mediated systems. Larger biomolecules, including insulin and transferrin, traverse the BBB via receptor-mediated transcytosis.^25^ Paracellular transport of hydrophilic molecules is tightly regulated by intercellular junctional complexes, including TJ proteins such as claudins and occludin, as well as adherens junction proteins.^26^ These components collectively seal the intercellular space, resulting in high transendothelial electrical resistance and severely limiting passive diffusion between endothelial cells. In addition to these structural barriers, metabolic degradation presents another significant challenge.^26^ Enzymatic activity within the BBB can degrade therapeutic agents, thereby restricting their passage and negatively affecting their distribution and retention within the brain.^27^

### 2.1 ATP-Binding Cassette Transports in BBB

ATP-binding cassette (ABC) transporters constitute a large family of membrane proteins that mediate the active transport of a wide range of substrates across the lipid bilayer of ECs, thereby contributing to the protective function of the BBB.^28^ These transporters are classified into seven subfamilies (ABCA–ABCG) based on their structural organization and sequence homology.^29^

Structurally, ABC transporters are composed of two transmembrane domains (TMDs), which are responsible for substrate recognition and translocation, and two cytoplasmic nucleotide-binding domains (NBDs) that bind and hydrolyze ATP.^30^ The transport process is driven by the hydrolysis of ATP, typically requiring two ATP molecules to translocate a single substrate against its concentration gradient across the cell membrane. This mechanism facilitates the movement of substances both into and out of cells, as well as between intracellular compartments (Figure 2).^31^

**Figure 2:**
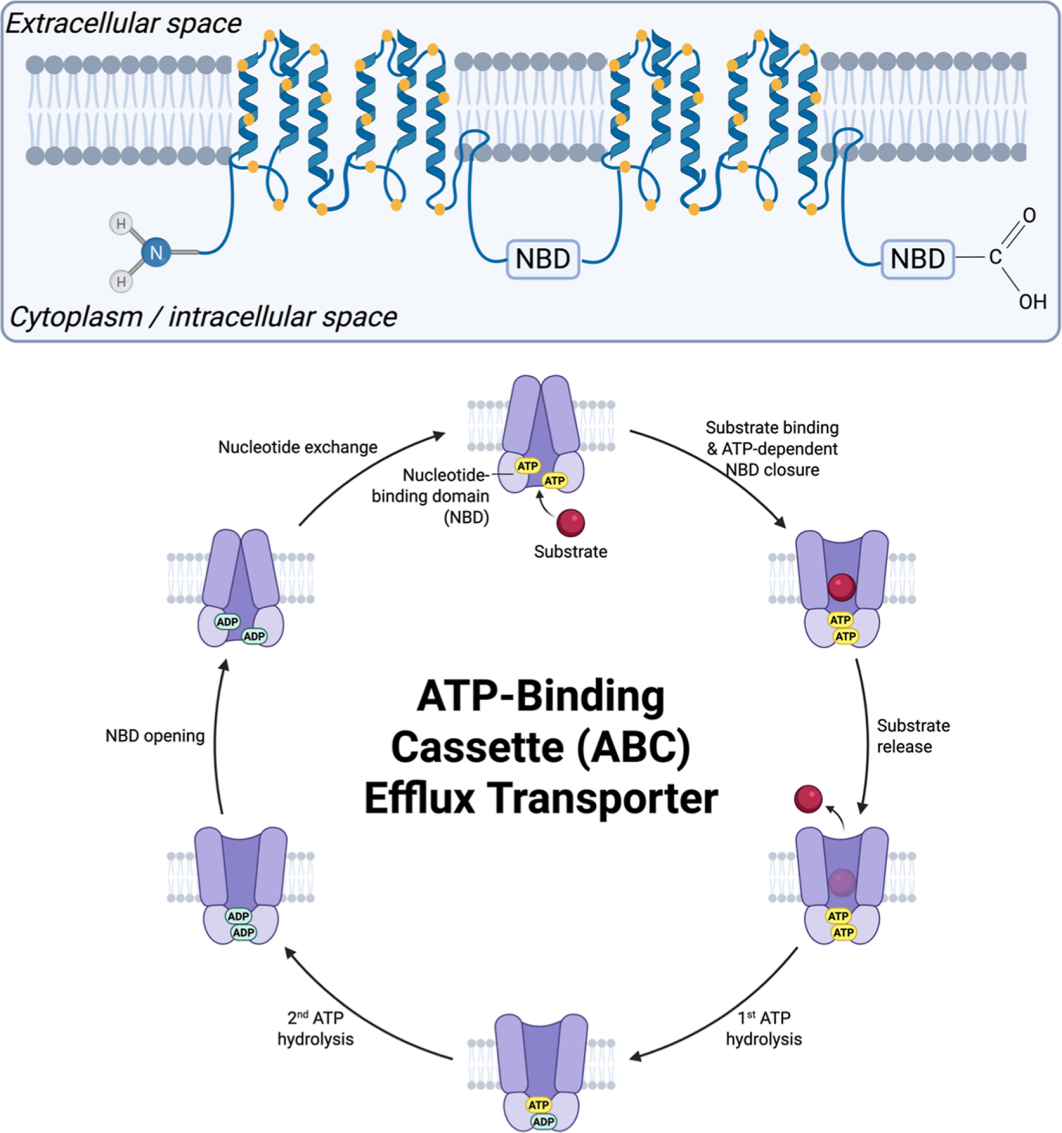
ATP-Binding Cassette (ABC) efflux transporter. The functional domains of ABC transporters are embedded within the membrane and extend across both the intracellular and extracellular sides, utilizing intracellular ATP to export substrates from the cytoplasm and protect cells from toxins. Created using www.biorender.com.

Within the BBB, ABC transporters are predominantly expressed on the luminal surface of endothelial cells, with comparatively lower expression levels observed in astrocytes, microglia, and neurons.^32^

#### 2.1.1 *P*-Glycoprotein (*P*-gp) Transporter

*P*-glycoprotein (*P*-gp) is a key ABC transporter belonging to the ABC subfamily B member 1 (ABCB1), also known as multidrug resistance protein 1 (MDR1).^33–35^ It plays a critical role in limiting drug accumulation in the brain by actively effluxing a wide range of therapeutic agents across the BBB. Two primary mechanisms have been proposed to explain *P*-gp-mediated efflux. The “vacuum cleaner” model suggests that *P*-gp captures substrates from the cytoplasmic aqueous phase and actively transports them across the lipid bilayer into the extracellular space.^36^ In contrast, the “flippase” (or “floppy”) model proposes that substrates initially partition into the lipid membrane, after which *P*-gp interacts with them within the hydrophobic core and translocates them by flipping from the inner to the outer leaflet of the membrane, ultimately releasing them into the extracellular aqueous environment.^37^ In vitro evaluation of *P*-gp substrates is a crucial step in CNS drug discovery, as failure to account for *P*-gp-mediated efflux can lead to drug resistance and a lack of therapeutic efficacy. Notably, many *P*-gp substrates are amphipathic in nature.^38^

Various strategies, including the use of *P*-gp inhibitors, have been explored to overcome this barrier and enhance drug delivery across the BBB. However, most of these approaches have been unsuccessful in clinical settings, largely due to issues related to toxicity and lack of selectivity.^39^

#### 2.1.2 Breast Cancer Resistance Protein Transporter

Breast cancer resistance protein (BCRP) is another member of the ABC transporter family. Structurally, it differs from *P*-gp in that it is a “half-transporter,” consisting of a single transmembrane domain and one nucleotide-binding domain.^40^ Despite this structural distinction, BCRP exhibits a broad substrate spectrum, with partial overlap with *P*-gp substrates, resulting in shared or “dual-substrate” specificity.^41^

Both *P*-gp and BCRP are coexpressed at the luminal surface of endothelial cells within the BBB, and their expression levels have been shown to increase with age, from fetal stages through to adulthood.^42^ Overexpression of these efflux transporters is strongly associated with the development of multidrug resistance.^43^ Furthermore, *P*-gp and BCRP can function cooperatively and compensate for one another in the active efflux of therapeutic agents, thereby significantly influencing drug pharmacokinetics and limiting CNS drug delivery.^44^

The restricted penetration of many CNS therapeutics is largely attributed to the activity of these transporters. For example, only approximately 5% of systemically administered methotrexate is able to cross the BBB.^45^ Notably, studies suggest that simultaneous inhibition of both *P*-gp and BCRP yields more pronounced improvements in drug brain uptake compared to inhibition of either transporter alone. This has been demonstrated in controlled studies using *P*-gp/BCRP knockout rodent models, where administration of radiolabeled (^14^C) tyrosine kinase inhibitors, such as the first-generation agent imatinib mesylate and the second-generation agent dasatinib, resulted in increased brain concentrations by approximately 1.25-fold and 10-fold, respectively.^46^

#### 2.1.3 Multidrugs Resistance Proteins

Multidrug resistance-associated proteins (MRPs) belong to the ABC subfamily C (ABCC), which comprises nine members in humans (ABCC1–ABCC9).^47^ Compared with other ABC transporters, the structure, function, and precise localization of MRPs at the BBB remain less well characterized.^48^

Structurally, certain members, specifically MRP4, MRP5, MRP8, and MRP9, are thought to resemble transporters from the ABCA and ABCB subfamilies, consisting of two transmembrane domains and two nucleotide-binding domains. In contrast, the remaining ABCC transporters possess an additional N-terminal transmembrane domain, resulting in a more complex architecture. However, the functional significance of this extra domain has yet to be fully elucidated.^49^

### 2.2 Limitation of Small Molecules

Small molecules (SMs) represent the largest class of therapeutics developed for CNS disorders, typically characterized by a molecular weight of less than 500 Da. These compounds access their targets primarily via passive diffusion and carrier-mediated transport mechanisms. Achieving an adequate free brain-to-plasma concentration ratio is essential for eliciting a therapeutic effect. To facilitate BBB penetration, SMs must possess favorable physicochemical properties, including a low polar surface area and minimal binding to plasma proteins such as human serum albumin.^50^

Adherence to Lipinski’s rule of five during CNS lead optimization is commonly employed to improve drug-like properties and enhance brain uptake. For instance, reducing the number of hydrogen bond donors decreases the likelihood of recognition by efflux transporters, while an optimal lipophilicity (*c*Log *P* ∼ 1–3) supports BBB permeability.^51^ However, fine-tuning lipophilicity remains challenging, as compounds must maintain a balance between sufficient membrane permeability and adequate aqueous solubility. Excessive lipophilicity (*c*Log *P* > 3) is often associated with increased toxicity and poor pharmacokinetic profiles.^52^ Moreover, strategies aimed at increasing lipophilicity can adversely affect other pharmacokinetic properties. For example, cyclization may enhance lipophilicity but can also increase molecular planarity, potentially promoting recognition by ABC transporters and thereby limiting brain penetration.^53^

SM therapeutics targeting cholinergic pathways, amyloid-β, and tau proteins are widely used in the management of neurodegenerative diseases. However, these agents are generally limited to symptomatic relief or modest slowing of disease progression and are typically effective only in the early to moderate stages of disease. Additional limitations include off-target receptor interactions, a high incidence of adverse effects, and cumulative toxicity associated with long-term administration.^54^

### 2.3 Limitations of Biologics and Macromolecular Therapeutics

Biologics are therapeutic products derived from biotechnology and living systems, and they are increasingly considered promising candidates for the treatment of CNS disorders due to their high target specificity.^55^ However, their clinical translation remains challenging. Factors such as large molecular size, poor absorption, susceptibility to enzymatic degradation, and potential immunogenicity contribute to high failure rates in clinical trials. In addition, limited potency is often observed as a consequence of inadequate penetration across the BBB.^56,57^

For example, recently approved antiamyloid monoclonal antibodies, such as lecanemab and donanemab, demonstrate limited therapeutic efficacy partly due to their poor BBB permeability following intravenous administration. To address this limitation, more invasive delivery approaches are often employed to bypass the BBB. However, these methods typically require repeated or prolonged administration and still result in relatively low concentrations reaching the target site. Furthermore, such approaches are associated with increased risks of adverse effects, high economic costs, and practical challenges that may impact patient compliance and acceptability.^58^ For instance, Lecanemab, like most therapeutic antibodies, exhibits limited intrinsic permeability across the BBB following intravenous administration. To enhance brain delivery, FUS has been used to transiently increase BBB permeability, thereby facilitating the entry of antibodies such as Lecanemab and Aducanumab into the brain parenchyma, where they bind amyloid-β (Aβ) species and promote their clearance.^59^ In contrast, Trontinemab employs a receptor-mediated transport strategy by targeting transferrin receptor 1 (TfR1), which is highly expressed on brain endothelial cells and naturally mediates iron transport across the BBB. Through engineering of a bispecific antiamyloid antibody capable of binding TfR1, enhanced transcytosis and improved central nervous system exposure may be achieved without substantially increasing systemic dosing.^60^

Macromolecules, including proteins, nucleic acids (e.g., RNA), carbohydrates, and other large biomolecules, offer high specificity and therapeutic potential compared with SMs for CNS disorders.^61^ Nevertheless, their large size and structural complexity significantly hinder their ability to cross the BBB. Additionally, the low percentage of injected dose per gram of brain tissue (% ID g^–1^) observed for many macromolecules is often attributed to rapid degradation and systemic clearance following administration.^62,63^

Consequently, invasive delivery strategies such as intrathecal injection or intracranial administration are frequently employed to enhance brain exposure. While these approaches can improve drug delivery to the CNS, they are associated with procedural risks and limitations that restrict their widespread clinical application.^64^

## 3 Safety Concern Associated Invasive Techniques

Numerous delivery strategies have been developed to enhance the transport of therapeutics into the brain parenchyma by overcoming or bypassing the restrictive effects of the BBB.^65^ Although some of these approaches have demonstrated improved drug concentrations within the CNS and reduced systemic toxicity, many remain invasive and are associated with significant risks to patients.^66^

One major category involves drug delivery via the cerebrospinal fluid (CSF). This approach relies on direct administration of therapeutics into the CSF, from which drugs subsequently enter the brain parenchyma through diffusion or convection. CSF-based delivery can be achieved through three primary routes targeting different anatomical regions: intrathecal injection into the lumbar region (lumbar intrathecal, LIT), administration into the cisterna magna (intracisterna magna), and intracerebroventricular (ICV) delivery via device implantation within the cerebral ventricles.^67^

ICV drug delivery requires surgical implantation of a catheter or reservoir system, making it highly invasive. This approach is associated with substantial risks, including neurological complications (reported in up to 33% of cases) and serious infections (approximately 27%).^68,69^ Additional practical challenges include catheter malposition, blockage, and leakage. Furthermore, the rapid turnover of CSF, renewed approximately 5× per day, necessitates the administration of large or repeated doses to maintain therapeutic concentrations. However, increased infusion volumes can elevate intracranial pressure and potentially result in neurological damage, thereby limiting the overall clinical efficacy of ICV delivery.^70^

Intrathecal (LIT) delivery also presents several limitations. The lumbar site of administration is anatomically distant from the brain, often requiring higher drug concentrations to achieve therapeutic levels in the CNS, which is particularly problematic for agents with narrow therapeutic windows.^71^ Repeated lumbar punctures are typically required for sustained treatment, leading to poor patient tolerability. While LIT has demonstrated high efficacy for certain drug classes, such as opioids, its effectiveness is considerably lower for biologics and macromolecules. Moreover, it is contraindicated in patients with infections or coagulation disorders.^72,73^

A second category of delivery strategies involves the controlled disruption of the BBB, most notably through focused ultrasound techniques. Although less invasive than direct CSF administration, these approaches still carry significant safety concerns.^74^ Focused ultrasound combined with microbubble-mediated delivery has been associated with potential risks, including long-term alterations in neuronal activity and neuroplasticity due to acoustic stimulation.^75^ Additionally, excessive heat generation during sonication may result in tissue damage, mechanical disruption, and even necrosis.^76^ The procedure may also involve the use of transdermal needles for microbubble administration, contributing to patient discomfort and procedural complexity.^77^

## 4 Peptide Vector as a Promising Strategy

Cell-penetrating peptides (CPPs) are a distinct class of short amino acid sequences capable of traversing cellular membranes without disrupting membrane integrity or function. They are also referred to as Trojan peptides, protein transduction domains (PTDs), or membrane-translocating sequences.^78,79^ Their favorable physicochemical properties, including low immunogenicity and enhanced bioavailability, have led to their widespread use as delivery vectors for transporting a variety of molecular cargoes across cell membranes via multiple uptake pathways.^80^ A specialized subset of these peptides, known as BBB-shuttling peptides, function as brain delivery vectors by exploiting their ability to cross the BBB, thereby enhancing the penetration of therapeutics into the central nervous system.^81^ In general, cell penetrating peptides (CPPs) are cationic; therefore, they can bind to anionic receptors such as heparan sulfate proteoglycans (HSPGs) and undergo receptor mediated endocytosis.^82^

Endogenous peptides such as bradykinin and glutathione naturally cross the BBB as part of physiological homeostasis. While this property has been leveraged for drug delivery, their lack of target specificity, due to widespread distribution in healthy tissues, limits their therapeutic precision.^83^ To address this, synthetic analogues and engineered derivatives have been developed to improve selectivity and efficacy. In this context, computational approaches are increasingly employed to screen and optimize peptide candidates, integrating in vitro and in vivo data to identify promising BBB-penetrating vectors.^83^

The present article focuses on highlighting key advancements, challenges, and advantages in the field, rather than attempting to provide an exhaustive overview. Accordingly, selected examples of peptide vectors are discussed to illustrate these aspects. A more detailed and comprehensive examination of CPPs in CNS drug delivery, particularly in relation to Alzheimer’s disease (AD) and Parkinson’s disease (PD), has been reported elsewhere.^84,85^

### 4.1 Angiopep-2

Naturally occurring proteins with inherent BBB permeability, such as apolipoproteins (Apo), have also been exploited to design brain-shuttling systems. Apo subfamilies B and E, for example, have been used in enzyme replacement strategies to facilitate the transport of therapeutic enzymes across the BBB, enabling the development of novel treatment approaches.^86^ A notable example is ANG1005, a conjugate comprising the human-derived peptide Angiopep-2 linked to three molecules of paclitaxel, which has demonstrated promising clinical efficacy in the treatment of brain metastases (NCT02048059).^87–89^

The Angiopep family comprises synthetic peptides derived from the Kunitz domain of various proteins. Among them, angiopep-2 has demonstrated high BBB penetration efficiency, an elevated brain volume of distribution, and a validated ability to transport therapeutic cargos across the BBB via LRP1-mediated transcytosis. Structurally, Angiopep-2 is a 19-amino-acid peptide, engineered from the Kunitz domain of LRP1 ligands^90,91^ (Figure 3).

**Figure 3:**
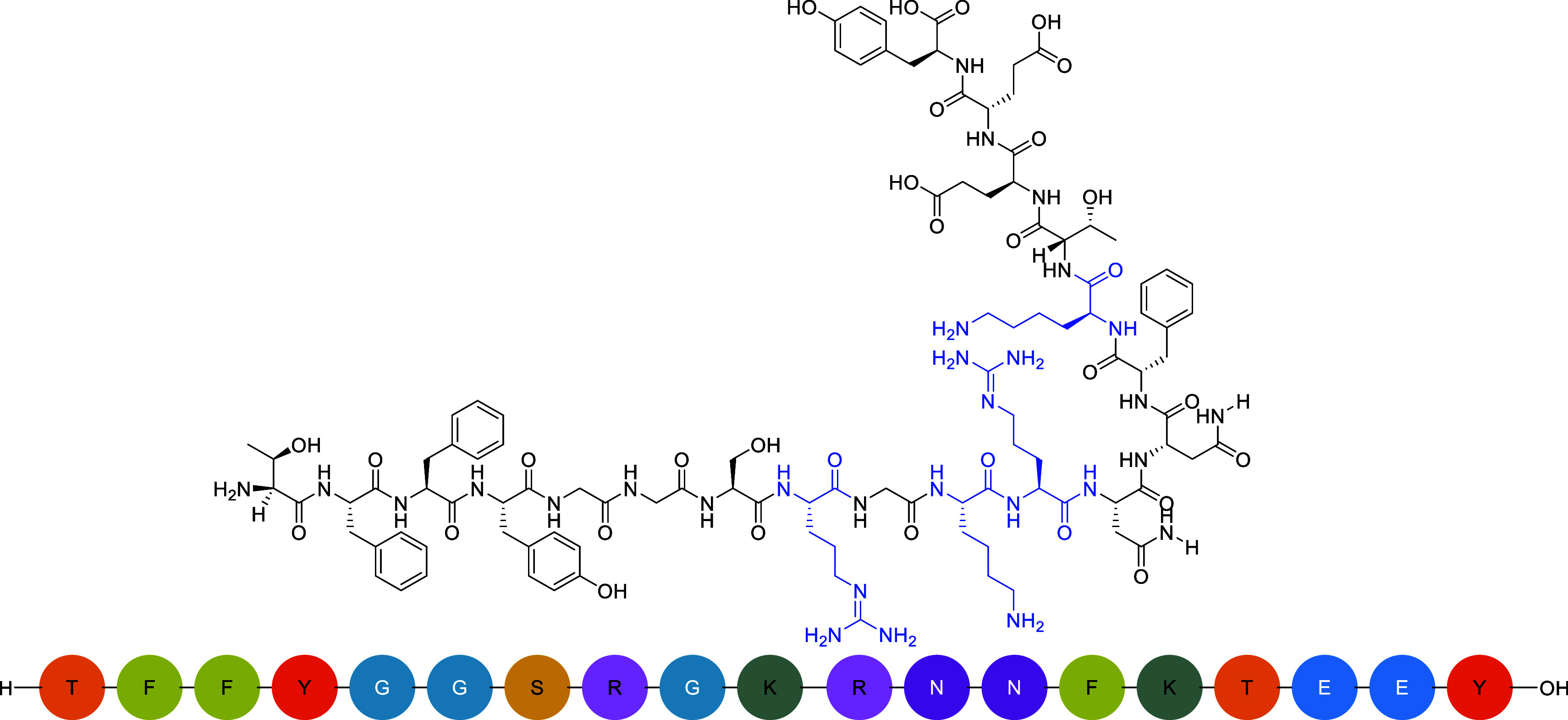
Chemical structure of angiopep-2. Blue, positively charged amino acid residues.

Recent computational molecular docking studies have further elucidated key features of the Angiopep-2-LRP1 interaction, revealing that the entire peptide sequence contributes to binding affinity, particularly through interactions with specific complement-type repeats (CRs) 17 and 56 of LRP1.^92^ Angiopep-2 has also been conjugated to small-molecule drugs, such as paclitaxel and morphine, to enable targeted delivery for brain metastases and neuropathic pain, respectively. In addition, it has been incorporated into nanoparticle and antibody-based drug delivery systems to enhance BBB permeability, target specificity, endosomal escape of cargo, and, in some cases, nuclear localization.^81^

### 4.2 TAT Peptide

TAT is a cell-penetrating peptide derived from the transactivator of transcription (Tat) protein of HIV-1. Its structure comprises 84–105 amino acids organized into six functional domains (Figure 4). TAT can be covalently conjugated to diverse cargo molecules, and its conjugation state and concentration significantly influence its cellular internalization pathway.^93^ TAT–cargo conjugates are primarily internalized via pinocytosis and clathrin- and caveolae-mediated endocytosis, whereas unconjugated TAT peptides predominantly enter cells through clathrin-mediated endocytosis.^93^

**Figure 4:**
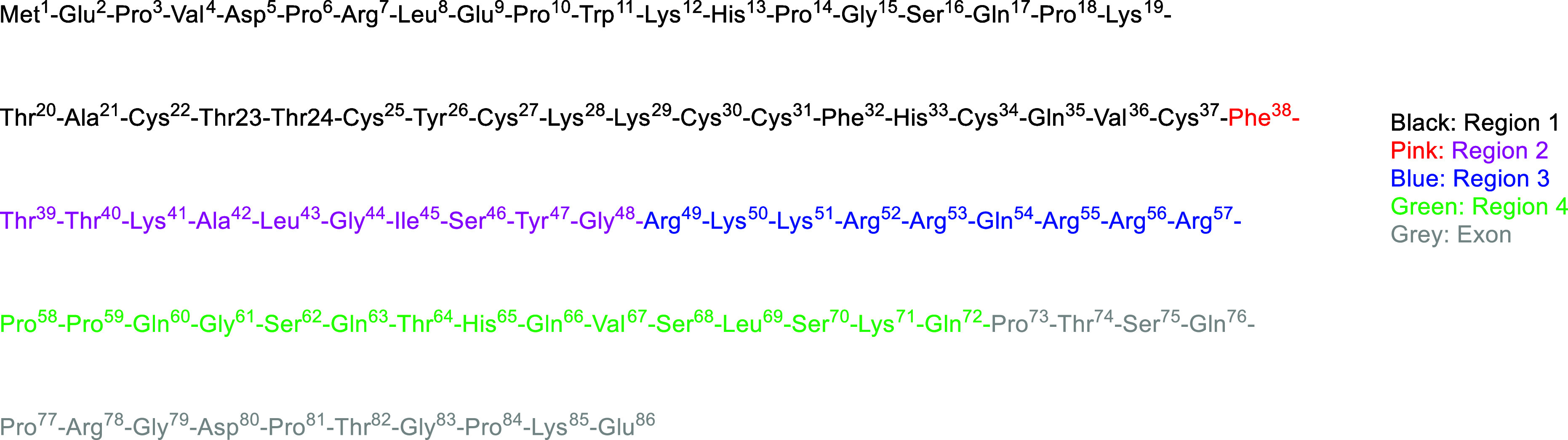
TAT sequence showing the tentative functional regions. Reproduced from ref 94 available under a CC-BY 4.0 license. Copyright 2022 by Othman Al Musaimi *et al.*.

In oncology, TAT conjugation to chemotherapeutic agents has been shown to prolong retention time, enhance receptor-mediated interactions, and improve brain uptake in both preclinical and clinical studies. For instance, TAT–doxorubicin demonstrated approximately 2-fold greater cytotoxic activity in murine colon adenocarcinoma cell lines compared with controls.^93^

In gene therapy applications, TAT-based peptides are widely employed to enhance cellular uptake of viral vectors by improving membrane penetration, target specificity, endosomal escape, and nuclear localization. Owing to their ability to traverse endosomal membranes following endocytosis, TAT peptides facilitate intracellular delivery of a wide range of therapeutic cargoes, although the precise escape mechanism remains incompletely understood.^95^ Nevertheless, this permeabilisation can affect cellular homeostasis, as TAT-mediated delivery has been associated in some cases with the release of functional components such as CHMP1B, Galectin-3, and TFEB, which are involved in membrane repair, organelle clearance, and biogenesis, respectively.

Several strategies have been developed to enhance TAT-mediated endosomal escape. For example, multivalent display of arginine-rich TAT peptides can increase local charge density, thereby improving endosomal disruption. Another optimized variant, dimeric TAT (dfTAT), formed via disulfide bonding, exhibits improved endosomal escape efficiency and enhanced cytosolic and nuclear localization. dfTAT has been shown to deliver a broad range of cargoes, including nanoparticles, small molecules, and macromolecules.^96^ Consistently, adenoviral vector modification with TAT has resulted in a 1–2-fold increase in transduction efficiency in coxsackie-adenovirus receptor–deficient cells.^96^

### 4.3 MTfp

Similarly, MTfp, a 12-amino acid peptide derived from the melanotransferrin (MTf) glycoprotein, is an emerging BBB shuttle corresponding to residues 460–471 of the full-length MTf protein (Figure 5).^97^ This peptide corresponds to the functional region responsible for iron transport across the BBB and has shown enhanced delivery of therapeutics in preclinical models.

**Figure 5:**
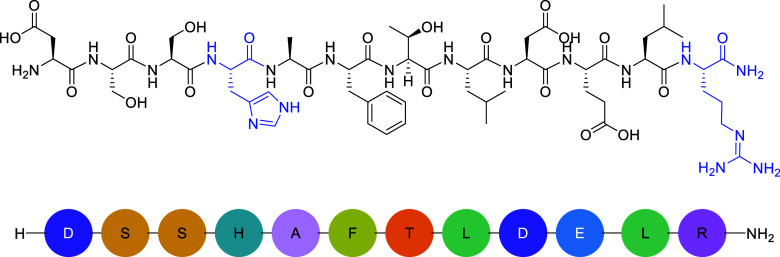
Chemical structure of MTfp. Blue, positively charged amino acid residues.

When conjugated to small interfering RNA (siRNA), MTfp improved outcomes in models of ischemic stroke, and when linked to interleukin-1 receptor antagonist, it demonstrated efficacy in neuropathic pain.^97,98^

### 4.4 RVG29

BBB-shuttling peptides have also been derived from viral proteins, notably the rabies virus glycoprotein (RVG), specifically residues 175–203 of its amino acid sequence (Figure 6). This region is capable of interacting with the α-subunit of the nicotinic acetylcholine receptor, facilitating entry into the central nervous system. A synthetic derivative, RVG29, 29-mer acid peptide, has been widely utilized as a delivery vector for small interfering RNA (siRNA) across the BBB. Owing to this inherent neurotropism, viral-derived peptides have become valuable tools for CNS-targeted drug delivery.^99^

**Figure 6:**
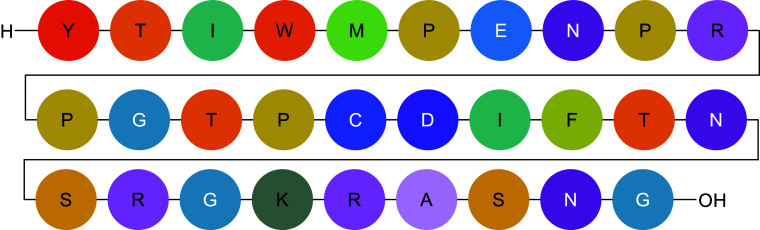
Amino acid sequence of RVG29. K, and R are the positively charged amino acid residues.

### 4.5 TD2.2

TD2.2 was developed as a putative human glial cell-specific targeting peptide based on a rational design framework. This approach was inspired by the infection mechanism of lymphocytic choriomeningitis virus (LCMV), an Old-World arenavirus that preferentially targets glial cells in the CNS through interactions between its surface glycoprotein GP1 and the extracellular domain of α-dystroglycan (α-D) (Figure 7).^100^

**Figure 7:**
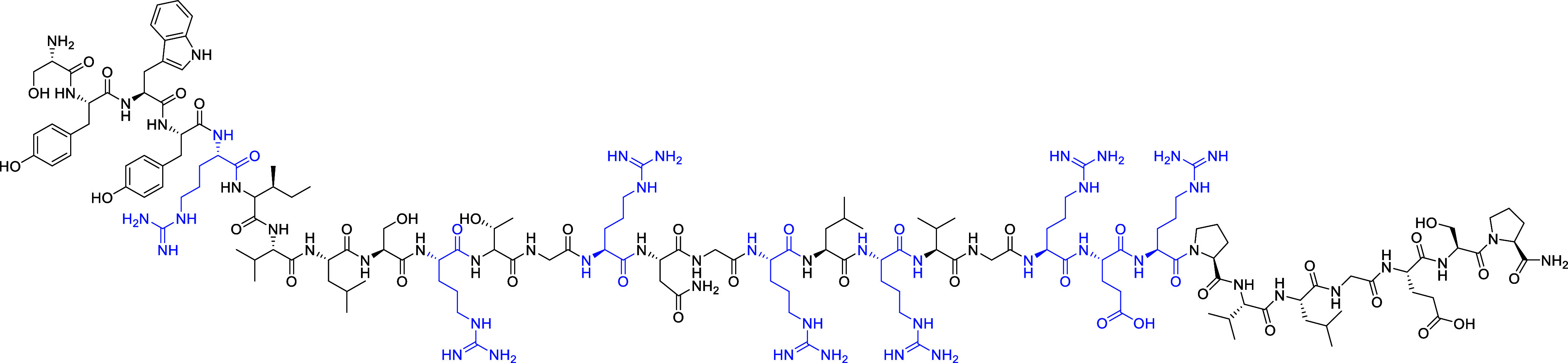
Chemical structure of TD2.2. Blue, positively charged amino acid residues.

In physiological conditions, α-D binds extracellular matrix proteins such as agrin and laminin-1/-2 with high affinity.^100^ Although the α-D residues involved in LCMV binding are known, the corresponding GP1 interaction motifs remain unclear. To address this, sequence alignment of GP1 with other α-D-binding proteins (e.g., laminin-2 and agrin) was employed to identify potential α-D-interacting domains, forming the basis for the design of a glial-targeting CPP.^100^

Subsequent screening demonstrated that TD2.2 selectively transduces glial cells, particularly immature oligodendrocytes, while showing minimal uptake in nonglial cells, including human neural cells and dermal fibroblasts.^100^ Time-lapse confocal microscopy confirmed intracellular trafficking of TD2.2 (fused to EGFP) in oligodendrocytes within 3–6 h of application. This selectivity suggests a promising strategy for targeting glia-associated diseases.^100^ Furthermore, the Al Musaimi group reported the first chemical synthesis of TD2.2 and developed a more proteolytically stable analogue.^101^ Using focused ultrasound combined with microbubbles, they achieved effective delivery of this analogue into the brain (data unshown).

### 4.6 MiniAp-4

In addition, venom-derived peptides have also been explored for their potential in CNS drug delivery. For example, MiniAp-4, derived from bee venom, has been developed as a BBB-shuttling peptide.^102^ Its structure, stabilized by disulfide bonds, confers enhanced resistance to metabolic degradation, making it a promising candidate for improving peptide stability and delivery efficiency across the BBB (Figure 8).^102^ MiniAp-4, a minimized and more permeable peptidomimetic derivative of apamin, 18-mer peptide amide, exhibits enhanced protease resistance and efficiently delivers a wide range of cargoes across the BBB in both human cell-based models and in vivo.^102^ Notably, it demonstrates strong brain-targeting capability while displaying reduced toxicity and immunogenicity compared to the native peptide.

**Figure 8:**
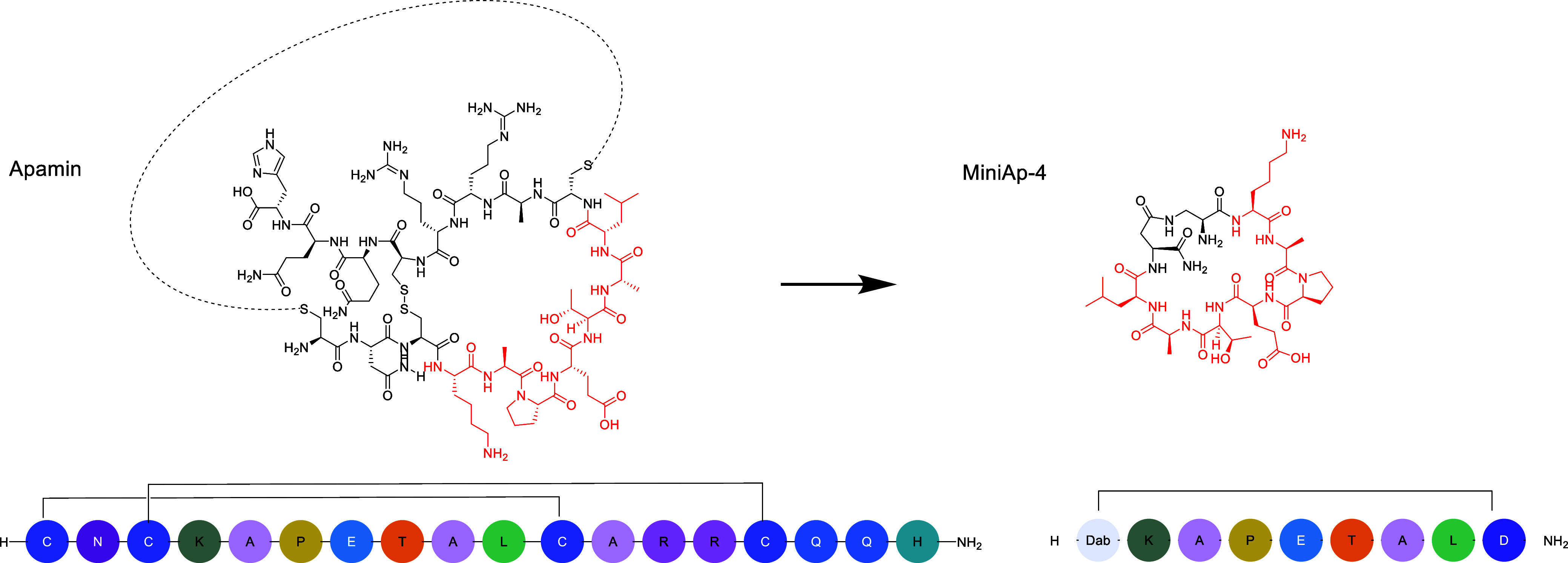
Chemical structures of Apamin and MiniAp-4. Red, mutual amino acid residues.

### 4.7 MiniCTX3

MiniCTX3, a monocyclic lactam-bridged peptidomimetic, effectively transports nanoparticles across endothelial cell monolayers, while MiniAp-4, a minimized form of apamin, enables efficient cargo delivery across the BBB in both in vitro and in vivo models (Figure 9).^103^ MiniCTX3 also enhances the permeability of both small and large cargoes in human BBB models, suggesting an active transcytosis mechanism that confers improved brain selectivity.^103^

**Figure 9:**
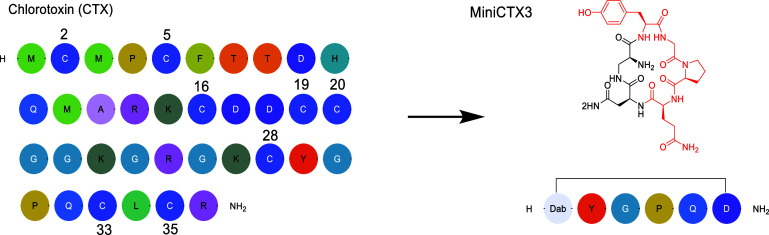
Chemical structures of chlorotoxin (CTX) and MiniCTX3. Red, mutual amino acid residues. Apamin has four disulfide bridges, Cys 2 and Cys 19; Cys 5 and Cys 28; Cys 16 and Cys 33; Cys 20 and Cys 35. Red, mutual amino acid residues.

Although native chlorotoxin (CTX), 36-mer acid peptide, can cross the BBB in vitro, its minimized analogues exhibit enhanced permeability while retaining protease resistance. Notably, MiniCTX3 improves the transport of gold nanoparticles (AuNPs) across a human BBB model. These peptides combine high BBB permeability with straightforward synthesis, offering a promising platform for brain-targeted delivery.^103^

### 4.8 p28

p28, a 28-amino-acid amphipathic peptide derived from azurin, represents an example of a peptide vector with potential antineoplastic and antiangiogenic activity^104–106^ (Figure 10).

**Figure 10:**
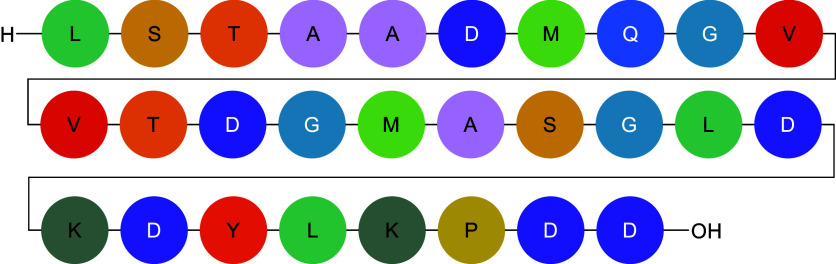
Amino acid sequence of p28. K is the positively charged amino acid residue.

Its preferential uptake by tumor and endothelial cells is thought to occur via caveolae-mediated endocytosis, followed by interaction with the tumor suppressor p53, leading to its stabilization and the induction of cell cycle arrest and apoptosis.^104^ In addition to its intrinsic activity, p28 can facilitate the delivery of macromolecules across a broad range of molecular weights.^107^ However, limitations in intracellular localization may reduce the efficacy of CPPs.^107^ Notably, p28 has been shown to undergo intracellular redistribution through modulation of endocytic pathways, and its combination with redistribution-inducing inhibitors has been reported to further enhance its anticancer effects (NCT01975116, NCT00914914).^108^

Collectively, these advances highlight the growing importance of peptide-based delivery systems in overcoming BBB-associated limitations. By integrating peptide engineering, computational design, and biological insights, BBB-shuttling peptides offer a promising strategy to enhance central nervous system drug delivery and expand the therapeutic potential of bioactive peptides.

## 5 Key Penetration Mechanisms

CPPs are capable of traversing cellular membranes and facilitating intracellular delivery of diverse molecular cargo. Their cellular uptake is governed by multiple mechanisms, reflecting their structural diversity and interaction with membrane components. Key penetration pathways include carrier-mediated transport, adsorptive-mediated transcytosis driven by electrostatic interactions with the cell surface, and receptor-mediated transcytosis (RMT), which involves specific ligand–receptor recognition. These mechanisms enable CPPs to overcome biological barriers and highlight their potential as versatile delivery vectors in therapeutic applications (Figure 11).

**Figure 11:**
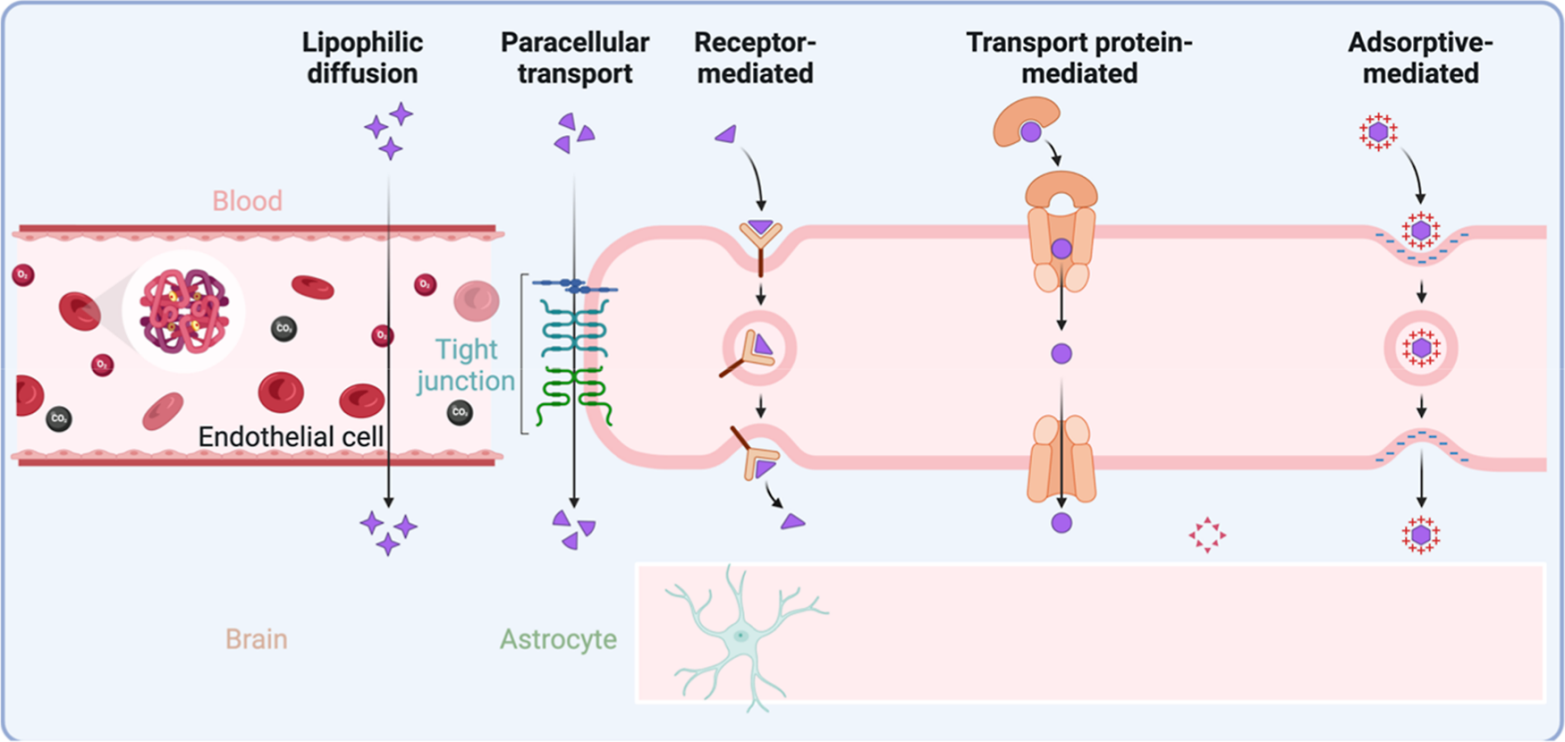
CPPs key penetration mechanisms. Created with www.biorender.com.

### 5.1 Carrier-Mediated Transport

Carrier-mediated transport (CMT) facilitates the translocation of essential nutrients, such as glucose and amino acids, across the BBB via highly specific and selective transporter proteins. Key examples include the glucose transporter GLUT1 and the large neutral amino acid transporter LAT1, both of which are expressed on the luminal and abluminal membranes of ECs. These transporters recognize and bind their respective substrates to form a substrate–carrier complex, which undergoes conformational changes to enable translocation across the BBB.^81^

This mechanism can be exploited for drug delivery, as certain therapeutics structurally mimic endogenous substrates. For instance, l-dopa and gabapentin share structural and physicochemical similarities with large neutral amino acids, allowing them to be transported across the BBB via LAT transporters.^109^

### 5.2 Adsorptive-Mediated Transcytosis

Adsorptive-mediated transcytosis (AMT) is a noncarrier and non- specific receptor-mediated transport mechanism across the BBB. It relies on electrostatic interactions between cationic substrates and negatively charged components of the endothelial cell membrane, such as proteoglycans. These interactions promote the formation of peptide–membrane complexes, which are subsequently internalized via endocytic pathways and transported across ECs.^110^ AMT is a concentration- and temperature-dependent, energy-requiring process involving endocytic machinery and electrostatic interactions with negatively charged cell surfaces. Although it shares features with receptor-mediated uptake, AMT is generally considered distinct from receptor-mediated transcytosis due to the absence of specific ligand–receptor recognition.^111–113^

A well-characterized example is the cationic peptide TAT, which has been employed as a BBB-shuttling vector to deliver β-galactosidase across the BBB in rodent models via the AMT pathway. However, a key limitation of AMT is its lack of cellular specificity within the brain, which may result in nonselective uptake and potential toxicity.^94,110^

### 5.3 Receptor-Mediated Transcytosis

Receptor-mediated transcytosis (RMT) is a highly selective transport mechanism that exploits receptors abundantly expressed on the Ecs of the BBB to facilitate drug delivery into the central nervous system. This process involves the binding of ligands or ligand-conjugated therapeutics to specific receptors, such as the transferrin receptor, insulin receptor, or low-density lipoprotein receptor-related proteins, which triggers receptor-mediated endocytosis and subsequent transcytosis across the endothelial layer.^114^

RMT offers significant advantages over other transport mechanisms due to its specificity and capacity for targeted delivery. By conjugating drugs or peptides to receptor-binding ligands, it is possible to enhance BBB penetration while minimizing off-target effects. However, limitations such as receptor saturation, competition with endogenous ligands, and potential alterations in receptor expression under pathological conditions must be considered when designing RMT-based delivery systems.^114^

#### 5.3.1 Transferrin Receptors

TfR1 is a highly expressed transmembrane protein at the BBB and is also overexpressed in various cancer cells, making it an attractive therapeutic target for central nervous system (CNS) disorders.^115^ TfR1 plays a critical role in iron transport, whereby iron-bound transferrin (holo-Tf) binds to TfR1 to form a holo-Tf-TfR1 complex that undergoes receptor-mediated endocytosis (Figure 12).

**Figure 12:**
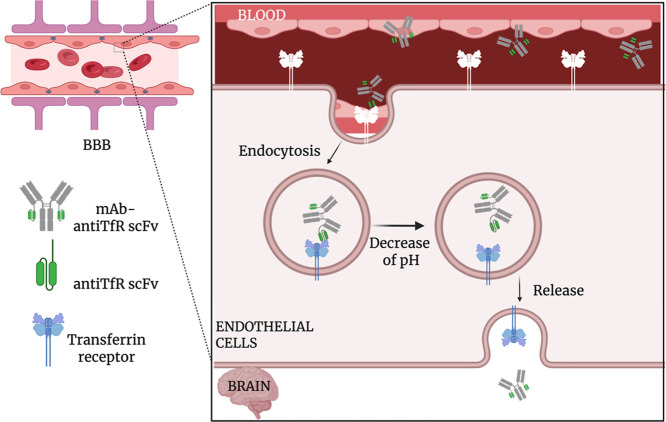
Transferrin receptor-1 (TfR1)-mediated transcytosis. Created with www.biorender.com.

Within endosomes, acidic pH promotes iron release, after which the receptor–ligand complex is recycled to the cell surface, where physiological pH facilitates dissociation into apo-transferrin and TfR1. These recycling and dissociation steps are key determinants of RMT efficiency across the BBB.^116^ Exploiting this pathway, transferrin-conjugated systems, such as transferrin-coated melittin nanoparticles (Tf-MeLioNs), have demonstrated effective brain delivery in AD models.^117^

#### 5.3.2 Low-Density Lipoprotein Receptor

The low-density lipoprotein receptor (LDLR) family comprises seven subfamilies of receptors involved in lipid transport and cellular uptake.^118^ Structurally, LDLR consists of an ∼850-amino-acid sequence organized into six functional domains.^119^ These receptors are highly expressed at the BBB and are overexpressed in certain brain cancers. Notably, LDL receptor-related protein-1 (LRP1) has been implicated in β-amyloid clearance and is considered a potential biomarker in AD.^120^ LDLR-mediated transport relies on ligand binding via cysteine-rich domains that recognize apolipoprotein substrates, enabling cholesterol transport and therapeutic targeting.^121^

#### 5.3.3 Insulin Receptor

The insulin receptor (IR), a member of the tyrosine kinase receptor family, mediates essential physiological processes including metabolism and growth. At the BBB, IR also contributes to receptor-mediated transcytosis. Structurally, it consists of two subunits linked by disulfide bonds to form a functional receptor complex.^122^ IR-targeting strategies have been explored for drug delivery, including the conjugation of lysosomal enzyme α-l-iduronidase (IDUA) to human IR monoclonal antibodies. Clinical studies (Phase I/II) have demonstrated stabilization of symptoms in patients with mucopolysaccharidosis type I (MPS I).^123^

### 5.4 Peptide Mediated Transport

Understanding the mechanisms underlying peptide–membrane interactions is critical for optimizing delivery efficiency and safety. Peptide internalization depends on multiple factors, including sequence, charge distribution, length, and concentration.^78^

#### 5.4.1 Cationic Peptides

Cationic peptides (CPs) are typically characterized by a high content of lysine and arginine residues, which play a crucial role in mediating their interactions with lipid membranes.^79^ These positively charged residues facilitate electrostatic interactions with negatively charged phospholipid components of the cell membrane.^124^

CPs can enter cells via direct translocation, a rapid, energy-independent process that occurs in a single step. This mechanism has been described by several models, most notably the “carpet-like” model and the membrane-thinning effect (Figure 13).

**Figure 13:**
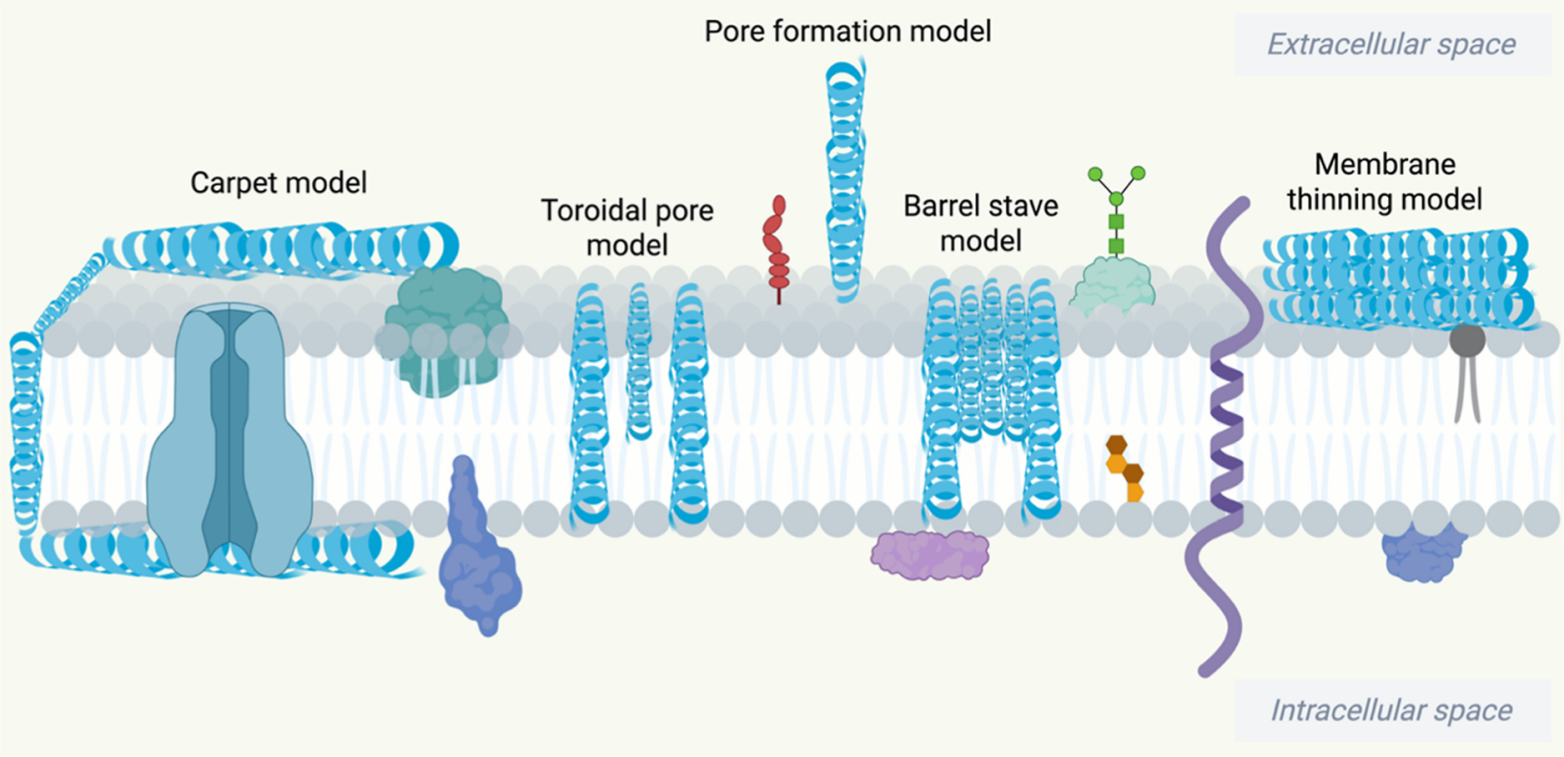
CPP-mediated peptide penetration models. Created with www.biorender.com.

In the carpet-like model, CPs align parallel to the cell surface, where their positively charged regions interact with negatively charged phospholipid head groups. Subsequently, the peptides undergo self-assembly into a carpet-like arrangement, in which hydrophobic segments insert into the lipid bilayer while hydrophilic regions remain exposed to the aqueous environment. This interaction induces membrane destabilization and structural rearrangement, ultimately facilitating peptide internalization.^125^ Illustrative examples of these models are presented in Figure 14. Although these peptides are not classified as CPPs, many, particularly antimicrobial peptides, exhibit similar membrane interaction and translocation mechanisms.

**Figure 14:**
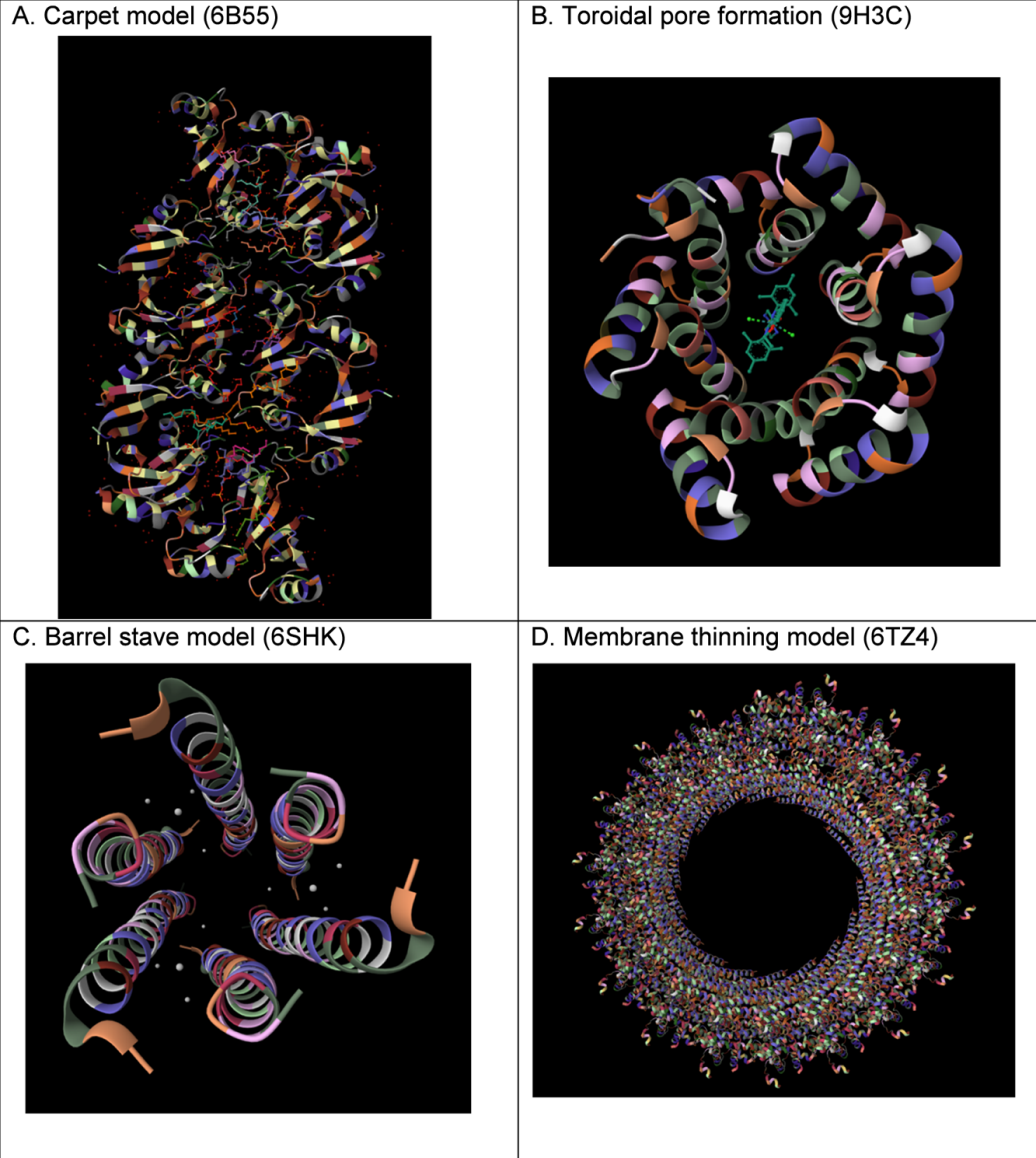
Illustrative examples for the peptide penetration models. (A) Carpet model, PDB ID 6B55;^126^ (B) Toroidal pore formation, PDB ID 9H3C;^127^ (C) Barrel stave model, PDB ID 6SHK. (D) Membrane thinning, PDB ID 6TZ4.^128^

#### 5.4.2 Amphipathic Peptides

Amphipathic peptides can also penetrate cell membranes through direct translocation involving the transient formation of pores. This mechanism is commonly described by the toroidal and barrel-stave models, which propose that interactions between amphipathic peptides and negatively charged cell membranes lead to the assembly of peptide bundles (α-helices or β-sheets). In these structures, the hydrophilic residues line the pore interior, while the hydrophobic surfaces interact with the lipid bilayer, facilitating membrane insertion and disruption (Figure 15).^129,130^

**Figure 15:**
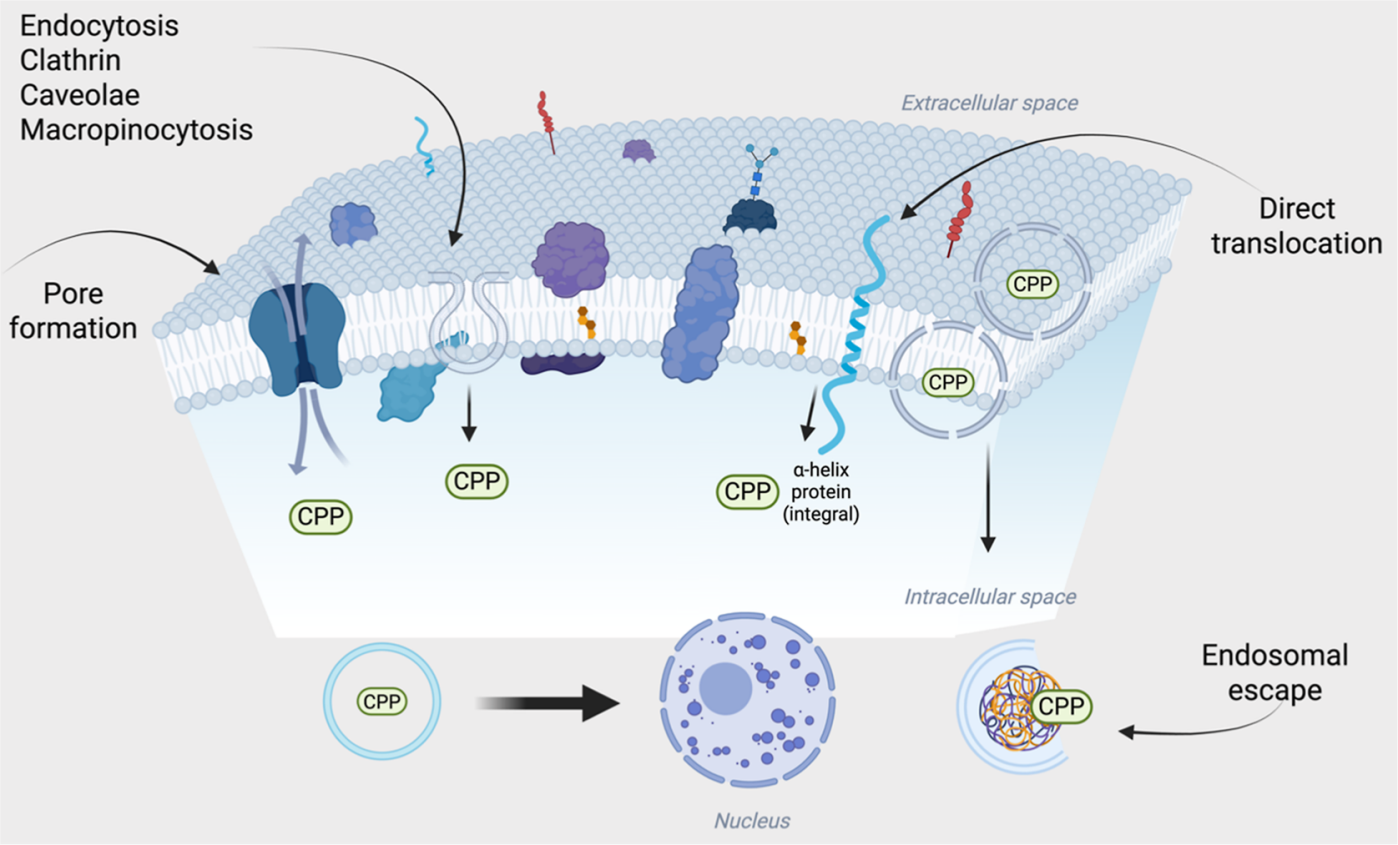
Mechanisms of CPP-mediated peptide penetration. Created with www.biorender.com.

At lower concentrations, peptides are primarily internalized via endocytosis, an energy-dependent, multistep process.^131^ During endocytosis, the plasma membrane envelops extracellular material to form intracellular vesicles that are transported into the cytosol. This uptake occurs through several pathways, including macropinocytosis, clathrin-mediated endocytosis, caveolae-mediated endocytosis, and clathrin- and caveolae-independent mechanisms. Following vesicle formation, the vesicles detach from the plasma membrane and may subsequently release their contents into the cytosol (Figure 15).^132^

## 6 Advantages, Limitations and Safety Concerns of Peptide Vectors

Peptides possess favorable biocompatibility and therapeutic potential due to their amino acid-based composition. They can penetrate cellular membranes through multiple pathways without significantly disrupting membrane integrity and are generally nontoxic and biodegradable. These properties make peptides safer delivery vectors compared with many conventional invasive and noninvasive delivery systems. In addition, peptides can compete with endogenous ligands and interact with a wide range of receptors in the human body.

Their physicochemical characteristics, including variable chain length and positive charge distribution, facilitate conjugation strategies and the formation of fusion proteins. Peptide size can also be engineered to enable condensation of proteins or DNA and the formation of nanoparticles for improved cellular uptake and gene expression. Positively charged residues enhance electrostatic interactions with both cell membranes and cargo molecules. Moreover, peptides typically exhibit low immunogenicity and are only toxic at relatively high concentrations, which are generally not required for delivery applications.^133^

Peptides also exhibit structural versatility, enabling self-assembly and stimulus-responsive conformational changes in response to environmental factors such as pH and temperature.^134–136^ These properties allow modulation of binding affinity toward receptors and cargo molecules, thereby supporting targeted delivery and controlled drug release. Furthermore, peptides are relatively straightforward to synthesize with high purity, and their structures are readily amenable to chemical modification.^137^

However, peptide-based systems also present limitations. Their susceptibility to proteolytic degradation leads to rapid clearance from biological fluids, which can reduce therapeutic efficacy.^138^ In addition, peptide-mediated internalization pathways, such as receptor-mediated transport, may inadvertently engage receptors expressed on nontarget cells, potentially resulting in off-target effects and toxicity. Moreover, peptide–cargo conjugation generates diverse complexes with distinct physicochemical properties, necessitating case-by-case evaluation of their mechanisms, efficacy, and safety. Detection of peptides in biological systems often relies on labeling techniques, which typically involve conjugation steps that may alter membrane-penetrating properties.^139^ From a manufacturing perspective, large-scale peptide production remains costly and technically challenging due to their complex chemical and biological characteristics. Solid-phase and liquid-phase peptide synthesis require extensive protecting-group strategies, making them suitable primarily for short or simple sequences, while longer or highly hydrophilic peptides are prone to aggregation and synthesis inefficiency.^140^ Additionally, sustainability concerns arise from substantial waste generation, including excess amino acids used during coupling reactions and large volumes of solvents required for purification and separation processes.^141^

Various chemical strategies have been employed to optimize peptide vectors by enhancing resistance to proteolytic degradation, extending systemic half-life, and improving binding affinity for more efficient and safe drug delivery. Among these approaches, hydrocarbon stapling and cyclization are commonly used to stabilize α-helical conformations or generate more conformationally rigid peptide structures.^94^ Stereochemical modification is another effective strategy for improving peptide performance. For example, comparisons between d-arginine- and l-arginine-based peptide–siRNA conjugates have shown distinct functional advantages. d-arginine-containing constructs exhibit significantly increased resistance to proteolytic degradation, whereas l-arginine variants demonstrate more efficient endosomal release of siRNA, highlighting a trade-off between stability and intracellular delivery efficiency.^142^

## 7 Conclusion

The BBB plays a critical protective role in shielding the brain from both endogenous and exogenous harmful substances, making effective drug delivery to the CNS highly challenging. Although many small molecules, biologics, and macromolecular therapeutics have demonstrated efficacy in in vitro models of CNS diseases, most have failed in clinical translation due to their inability to cross the BBB. While direct intracerebral injection approaches have been used clinically to improve CNS drug bioavailability, these methods are invasive and often associated with significant adverse effects.

In this context, peptide-based delivery systems have emerged as a promising noninvasive strategy to enhance CNS drug transport. Owing to their intrinsic physicochemical properties, peptides can traverse cellular membranes through multiple mechanisms. Their structural flexibility, ease of chemical modification, and capacity to conjugate diverse therapeutic cargos further contribute to their attractiveness as delivery vectors capable of facilitating transport across the BBB. Additionally, peptides with high affinity for specific receptors can enhance targeting specificity, thereby reducing systemic toxicity and off-target effects. Despite encouraging preclinical and clinical evidence supporting the efficacy of peptide-based vectors, several limitations remain that must be addressed to achieve safe and optimized CNS drug delivery.

Future research should prioritise improving endosomal escape, which appears to be a key rate-limiting step in delivery efficiency. Given the extreme sensitivity of the CNS, where many cells are irreplaceable and tightly regulated cellular networks are essential for function, it is crucial to carefully evaluate how peptide-mediated endosomal escape and cargo release affect cellular homeostasis. A deeper understanding of how modifications to cell-penetrating peptides influence these processes will be fundamental to minimizing cellular stress while maintaining delivery efficiency.
